# Tau Biomarkers in Dementia: Positron Emission Tomography Radiopharmaceuticals in Tauopathy Assessment and Future Perspective

**DOI:** 10.3390/ijms222313002

**Published:** 2021-11-30

**Authors:** Maria Ricci, Andrea Cimini, Riccardo Camedda, Agostino Chiaravalloti, Orazio Schillaci

**Affiliations:** 1 Department of Biomedicine and Prevention, University of Rome Tor Vergata, 00133 Rome, Italy; andreacimini86@yahoo.it (A.C.); riccardo.camedda@gmail.com (R.C.); agostino.chiaravalloti@uniroma2.it (A.C.); orazio.schillaci@uniroma2.it (O.S.); 2 Nuclear Medicine Section, IRCCS Neuromed, 86077 Pozzilli, Italy

**Keywords:** Tau tracers, first-generation Tau tracers, second-generation Tau tracers, tauopathy, Tau biomarkers, PET, Tau PET, Alzheimer’s disease, dementia, [^18^F]Flortaucipir

## Abstract

Abnormal accumulation of Tau protein is closely associated with neurodegeneration and cognitive impairment and it is a biomarker of neurodegeneration in the dementia field, especially in Alzheimer’s disease (AD); therefore, it is crucial to be able to assess the Tau deposits in vivo. Beyond the fluid biomarkers of tauopathy described in this review in relationship with the brain glucose metabolic patterns, this review aims to focus on tauopathy assessment by using Tau PET imaging. In recent years, several first-generation Tau PET tracers have been developed and applied in the dementia field. Common limitations of first-generation tracers include off-target binding and subcortical white-matter uptake; therefore, several institutions are working on developing second-generation Tau tracers. The increasing knowledge about the distribution of first- and second-generation Tau PET tracers in the brain may support physicians with Tau PET data interpretation, both in the research and in the clinical field, but an updated description of differences in distribution patterns among different Tau tracers, and in different clinical conditions, has not been reported yet. We provide an overview of first- and second-generation tracers used in ongoing clinical trials, also describing the differences and the properties of novel tracers, with a special focus on the distribution patterns of different Tau tracers. We also describe the distribution patterns of Tau tracers in AD, in atypical AD, and further neurodegenerative diseases in the dementia field.

## 1. Introduction

The erroneous folding of some structural proteins of neurons is associated with several neurodegenerative diseases. These are progressive and irreversible processes with variable neurological disturbances (e.g., language, visuospatial orientation and problem-solving skills’ impairment, loss of memory, changes in behavior) responsible for cognitive impairment and dementia, such as Alzheimer’s disease, tauopathies, synucleinopathies and prionopathies [1,2,3].

### 1.1. Epidemiology

Alzheimer’s disease (AD) is the most common form of dementia, characterized by abnormal protein aggregates and accounting for 60–80% of the total dementia cases. As the leading cause of dementia, AD is considered one of the fastest-rising diseases among the leading causes of death worldwide [4,5,6]. In 2020, there were over 50 million people living with AD worldwide and epidemiologic data from the last decade show a sharp growth of AD incidence, owing to the older population’s constant growth in size. Both genetic and environmental risk factors play a role in AD, but age is the greatest contributor. AD can be classified by when it manifests: early-onset AD (EOAD) occurs before age 65, while late-onset AD (LOAD) occurs after age 65 and accounts for over 95% of cases [7]. Most AD cases are sporadic, but the identified AD genetic risk factors are related to the APO-E genotype [8,9]. Alzheimer’s disease is characterized by a long asymptomatic preclinical phase, known to begin 20 years or more before clinical onset, with changes in the brain unnoticeable to the people affected [10,11,12]. To develop targeted intervention strategies for AD treatment, we first need to focus our resources on finding early markers of brain changes that happen before the onset of cognitive impairment.

### 1.2. Neuropathology

There are two types of major neuropathological changes of the brain with AD. The first is from upregulation of a certain element, and consists of accumulation of soluble β-amyloid (Aβ) peptide in parenchymal plaques, vascular deposits, intraneuronal neurofibrillary tangles (NFT), neuropil (NT) strands and dystrophic neurites made up of protein associated with misfolded and hyperphosphorylated microtubules. The others are instead represented by downregulated or repressed elements, such as the loss of neurons, neuropil and synaptic elements and progressive and selective cerebral atrophy [13,14,15,16]. The accumulation of β-amyloid plaques outside neurons and the accumulation of Tau tangles inside neurons, known since Aloise Alzheimer described them more than 100 years ago, are cardinal features for AD, required for its pathological diagnosis. Their appearance precedes the clinical onset by years [10,11,17]. Senile plaques are principally made of the extracellular accumulation of Aβ40 and Aβ42 peptides that results from the abnormal processing of amyloid precursor protein (APP) by the β and γ secretases, with Aβ sheets that are resistant to degradation. The increased concentration of Aβ42 promotes the formation of oligomers, which have neurotoxic properties, by interfering with neuron-to-neuron communication at synapses. These oligomers tend to gather around the grey matter and meningeal and cerebral vessels in AD. Co-localized with neuronal debris and activated microglia and astrocytes, they first appear in the frontal, temporal and occipital lobes of the neocortex. They spread throughout neocortical areas as well as the hippocampal formation and entorhinal region, and eventually spread further throughout the cerebral cortex to the striatum and thalamus [18,19,20,21,22,23,24].

### 1.3. Tau Deposition

Neurofibrillary tangles are fibrillary intracytoplasmic structures in neurons made of a protein called Tau. Tau protein has the main function of stabilizing axonal microtubules, and usually, it presents a certain number of phosphate molecules attached to it. In AD, the phosphorylation mechanics of Tau are altered, and this alteration leads to an abnormal increase of the phosphorylation and consequent detachment of Tau molecules from the microtubules [25,26,27]. Altered Tau proteins tend to assemble, forming filamentous structures known as paired helical filaments, which in turn, aggregate in insoluble neurofibrillary tangles [25,26]. Tau tangles impede the transport of nutrients and other essential molecules inside neurons. Like plaques, they spread throughout the brain as the disease progresses, beginning near the entorhinal cortex, spreading to the hippocampus and finally progressing to cover the cerebral cortex [28,29]. The progressive spread of neurofibrillary tangles underlies Braak′s staging of Alzheimer′s disease. Introduced in 1991, it consists of six stages that describe the progressive involvement of the brain in the advancement of the pathology. Stages I and II are defined by the presence of neurofibrillary tangles mainly limited to the transentorhinal region of the brain; stages III and IV are associated with involvement of the limbic regions; stages V and VI are when the pathology is widespread and there is wide neocortical involvement [16,30,31]. At the same time, hyperphosphorylated Tau protein gives rise to neuropil threads, which are abnormal structures located chiefly in distal dendrites, made up of straight and paired helical filaments [27].

### 1.4. Biomarkers in Dementia

Both amyloid plaques and Tau tangles are associated with neuronal damage and loss although the specific mechanism remains uncertain. Some results show that the density and neocortical spread of NFTs correlate better with neurodegeneration and cognitive decline in AD patients, despite amyloid disease temporally preceding Tau disease [32,33,34]. In addition to the Aβ and Tau pathology, other processes, such as synaptic dysfunctions and microglia-mediated inflammation, also play an important role in AD pathogenesis and may correlate with cognitive decline [35]. Levels of Aβ42, Tau and P-Tau in the cerebrospinal fluid (CSF) are correlated with senile plaques and neurofibrillary tangles: low levels of Aβ42 and high levels of Tau and P-Tau are typical but not restricted to AD, though these changes differentiate patients with AD from normal subjects or patients with other neurologic conditions with high accuracy [36,37]. Moreover, a direct association between subjective cognitive decline and decreased Aβ42 and increased Tau or P-Tau has been studied in patients with mild cognitive impairment (MCI) who later will develop AD [38,39]. There is a significant relationship between quantities of amyloid plaques and levels of CSF biomarkers and an inverse correlation between the total AB load and CSF Aβ42 level in the brain [39], suggesting that levels of these fundamental CSF molecules could correlate, in some circumstances, with the prediction, disease progression and severity of cognitive decline [40,41].

Tau and P-Tau proteins are considered markers of neurodegeneration; patients with AD show a 300% rise in t-Tau levels when compared with elderly unaffected subjects. However, these data are not pathognomonic for AD as the increase in concentration is also found in other pathologies characterized by neuronal damage with an inflammatory, traumatic or degenerative etiology. A study has shown that Tau and P-Tau are associated with a more aggressive form of AD, with very high values associated with higher mortality [42].

New NIA-AA criteria for Alzheimer’s disease place a great emphasis on the role of biomarkers, which may be essential for determining the Alzheimer’s etiology of the clinical picture. They represent a detachment from the past and a real novelty in relation to the traditional assumptions that substantially founded the diagnosis of AD on a clinical process aimed at excluding other possible causes of dementia and on the recognition of the disease at the time of its full-blown clinical manifestation. It has now been established that Alzheimer′s disease should be defined as a biological process identified by biomarkers in living people. New guidelines for the diagnosis of AD in clinical research take into account: biomarkers of Aβ plaques (labeled “A”) such as low CSF Aβ42 and cortical amyloid PET ligand binding; biomarkers of fibrillar Tau (labeled “T”), such as elevated CSF p-Tau and cortical Tau PET ligand binding; biomarkers of neurodegeneration or neuronal injury (labeled “(N)”), such as elevated CSF Tau, [^18^F]FDG PET hypometabolism and atrophy on MRI [43]. The presence of biomarkers “A” and “T” determines AD as a continuum (alterations of different proteins define the state of disease), while “N” biomarkers are not specific to Alzheimer′s disease but are used to stage its severity [44]. The measurement of Aβ peptides and Tau levels in the CSF could be used as a complementary tool in the diagnosis and monitoring of AD, as stated by the European Medicines Agency [45,46]. This represents a less expensive method of evaluation, but it is invasive and carries the risk of adverse effects and discomfort associated with a lumbar puncture. In the context of non-invasive methods, modern imaging techniques play an important role, helping either to identify patients who are at risk of developing AD or to monitor disease progression, or both [47].

### 1.5. PET Imaging in Dementia

PET (positron emission tomography) imaging is known for its high sensitivity and the ability to visualize and quantify physiological activities at the molecular and cellular levels [48]. It, therefore, represents an important method in the field of research for the discovery and development of new drugs able to monitor disease progression and the interaction of ligands with their targets. Several tracers can be applied to the neurodegenerative study field that range from the widely used [^18^F]FDG to novel Tau tracers that allow the assessment of the in-vivo distribution of Tau deposition. 

### 1.6. Purpose of This Manuscript

In this review article, we focus on the usage of PET imaging in the assessment of tauopathy. Particularly, we describe the findings concerning the correlation between the brain glucose metabolism (evaluated using [^18^F]FDG imaging) and Tau deposition (evaluated by CSF biomarkers). However, the main aim of this review is to provide an update on the role of Tau tracers in the evaluation of the Tau-pathology in the field of AD research. We do so by also focusing on the bio-distribution of Tau tracers in the brain regions, providing a comprehensive description of the actual knowledge about the Tau PET distribution pattern and also considering aspects that may influence the in-vivo distribution of Tau tracers in the brain. 

## 2. Tauopathy and Brain Glucose Metabolism

[^18^F]FDG PET plays a major part in clinical assessment for cancer diagnosis, but its first application was brain imaging [49]. Nowadays, it is considered the most common functional brain imaging method in the study of the Alzheimer′s pathology and dementia. In people with dementia, brain hypometabolism detected with [^18^F]FDG PET is a marker of neurodegeneration. PET imaging measures regional glucose consumption related to local glutamatergic synaptic and astrocyte activity. [^18^F]FDG PET allows us to identify areas of hypometabolism and evaluate their extent, information that reflects neuronal dysfunction [50,51].

Numerous studies have taken place over the years and agreed on finding a constant reduction in brain glucose metabolism in the precuneus, posterior cingulate, parietal and temporal cortex in clinically affected patients. Similarly, glucose hypometabolism extends to the frontal cortex and the entire brain as the disease becomes more severe. Glucose metabolism is usually kept safe in the primary motor sensory cortices, primary visual cortices, striatum, thalamus and cerebellar hemispheres [52,53,54,55].

PET brain imaging with [^18^F]FDG is also studied in relation to levels of CSF biomarkers (t-Tau, p-Tau and Aβ_1−42_ amyloid peptide), to identify possible correlations.

The values of CSF biomarkers and [^18^F]FDG PET imaging of a group of symptomatic subjects with suspected AD was studied to evaluate the concordance and relationship between the two types of biomarkers. They appear to have a complementary value in diagnosing AD, according to a moderate level of concordance. CSF Aβ_1−42_ and t-Tau values can help to predict the glucose metabolic status in the patient′s cerebral cortex [56].

Strong agreement was also found between the results of [^18^F]FDG-PET and CSF t-Tau in patients with moderate AD, supporting the idea that both should be considered markers of neurodegeneration and could replace each other as supportive diagnostic tools in patients with suspected moderate-to-severe AD dementia. This consideration is not true in subjects with early-stage disease [57].

AD is a clinical continuum that includes AD dementia, prodromal AD (MCI) and preclinical AD (cognitively normal). Knowing the progression of the disease through these three phases and the speed with which it manifests is fundamental, along with considering the development of new drugs able to intervene early on in the disease progression. Brain [^18^F]FDG-PET imaging and the CSF p-Tau/Aβ42 ratio may predict both MCI progression to overt AD dementia and the conversion time. The positivity of both biomarkers in patients with MCI suggests a probable short-term development of AD dementia [58].

Decreased levels of Aβ_1−42_ in CSF are associated with extensive cortical dysfunction, while alterations in t-Tau and p-Tau are accompanied by more selective cortical metabolic alterations mainly involving the cingulate cortex [59].

**Figure 1:**
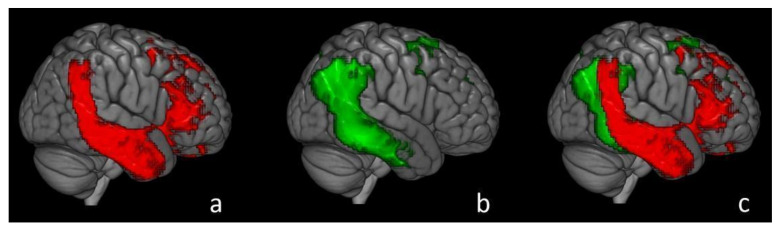
A figure published in a previous paper of our research group, focused on the comparison of brain glucose metabolism and T-Tau level in CSF in AD. Three-dimensional brain rendering showing in (**a**) the cluster obtained in SPM regression analysis of T-Tau in AD (red). In (**b**), we show the cluster obtained from the comparison of HC and AD subjects (hypometabolism in AD, green). In (**c**), both clusters are merged in a single image, showing the overlap of the temporal and parietal cortices (see text). AD: Alzheimer’s disease; HC: healthy controls. Image source: Brain metabolic correlates of CSF Tau protein in a large cohort of Alzheimer’s disease patients: A CSF and [^18^F]FDG PET study. A. Chiaravalloti et al./Brain Research 1678 (2018) 116–122 [60].

A study of our research group conducted on over 100 patients with a recent diagnosis of AD showed a significant negative correlation between CSF levels of t-Tau and cerebral metabolism assessed with [^18^F]FDG in the right temporal, parietal and frontal lobes, without further significant correlation with the values of the other CSF biomarkers. This indicates that t-Tau deposits in the brain are correlated with temporal, parietal and frontal hypometabolism in Alzheimer′s disease. Yet, a significant negative relationship between t-Tau and [^18^F]FDG uptake was found in a wide cluster that included in decreasing order the right temporal, right frontal and right parietal cortices (Figure 1) [60]. 

The study of CSF biomarkers can lead to the identification of subgroups of patients with Alzheimer′s disease, and [^18^F]FDG-PET allows the evaluation of the cerebral metabolism of the different subgroups.

Considering a group of patients attributable to the probable early onset of AD (EOAD), CSF biomarkers allow us to identify three clusters: the first with low levels of Aβ42 and normal levels of t-Tau and p-Tau; the second with low levels of Aβ42 and high levels of t-Tau and p-Tau; and finally, the third with very high levels of t-Tau and p-Tau and low-normal levels of Aβ42. The second and third groups show more marked frontal hypometabolism than the first group, and glucose hypometabolism here correlates significantly negatively with CSF Tau levels. At the same time, the first and second groups show more marked glucose hypoactivity in the left occipitotemporal region than in the third cluster, with a significant positive correlation with CSF Aβ42 levels [61].

[^18^F]FDG PET brain imaging was also used to compare the brain metabolism of SNAP (suspected non-Alzheimer′s pathophysiology) patients with that of AD patients and healthy controls, using CSF biomarkers to distinguish SNAP and AD patients. SNAP subjects present normal levels of amyloid-β protein markers (A−) and abnormal levels of markers of neurodegeneration or neuronal injury (N+). In comparison with SNAP subjects, it was seen that AD patients show significant hypometabolism in a large cortical area, with involvement of the right temporal, parietal and frontal lobes. The comparison between healthy controls and AD subjects showed significant glucose hypometabolism in the parietal, limbic and frontal cortices; a less evident reduction in glucose metabolism was observed in the same areas when comparing SNAP patients to healthy controls. Metabolic alterations in SNAP patients are, therefore, milder than in AD patients, resulting in a partial overlap of the metabolic pattern that means brain [^18^F]FDG-PET imaging is not the ideal method to effectively distinguish these two clinical entities [62,63].

PET imaging with [^18^F]FDG has also been used to study the relationship between hypothalamic changes in AD patients and sleep disturbances in relation to AD CSF biomarkers [64]. As demonstrated by post-mortem studies, Alzheimer′s disease affects the hypothalamic nuclei responsible for the sleep-wake rhythm. PET imaging has documented a significant reduction in hypothalamic glucose metabolism in patients with AD, in association with a marked alteration of nocturnal sleep with a negative correlation between [^18^F]FDG absorption and the t-Tau/βamyloid42 ratio, an index of more marked neurodegeneration.

A further paper investigated the relationship of Tau tracers’ distribution with glucose metabolism and [^18^F]FDG absorption, suggesting that the Tau pathology overlapped with areas of hypometabolism on the [^18^F]FDG PET in the brains of AD patients [65].

Various biomarkers are available to support the diagnosis of neurodegenerative diseases in clinical and research settings. Among the functional imaging, [^18^F]FDG imaging provides valuable information. The European Association of Nuclear Medicine and European Academy of Neurology recommendations support the use of [^18^F]FDG in the early and differential diagnosis of the main neurodegenerative disorders, and in semi-automated assessment to assist visual reading. However, the use of [^18^F]FDG-PET is not supported in any of the preclinical conditions assessed by a panel [66]. This reflects the capability of [^18^F]FDG imaging in detecting patterns and the extent of neuronal dysfunction in the case of cognitive decline, but it also reflects the importance of improving our knowledge about the potential for assessment of preclinical conditions and of the entire clinical spectrum of AD and dementia. Concerning tauopathy, which represents a crucial aspect in the physiopathology of dementia, further tools are needed in order to assess the role of Tau accumulation in AD and other neurodegenerative diseases. All the above-mentioned papers correlated the Tau accumulation (assessed by fluid biomarkers) and the cortical dysfunction (assessed by [^18^F]FDG distribution) in cortical regions with heterogeneous results. Even if it seems obvious that a certain degree of cortical dysfunction can be detected in the case of claimed neurodegeneration (assessed by Tau biomarkers or others), the possibility to correlate specific regional hypometabolic patterns to tauopathy may support the clinical practice in symptomatic patients. However, a direct assessment of tauopathy by Tau-PET imaging may improve not only the clinical practice but also the research field, especially considering the preclinical conditions. Therefore, an accurate in vivo Tau assessment may improve our knowledge about the neuro-physiopathology of AD and dementia, and subsequently, may provide novel insight into the dementia field with a potential crucial impact on its management and treatment.

## 3. Tau PET Imaging

Tau PET imaging is promising to serve as a biomarker to support differential diagnosis and monitor disease progression in Alzheimer’s disease and other neurodegenerative diseases. The clinical and scientific interest in Tau PET tracers is rapidly growing, with an increasing number of papers aiming to provide novel insight into the first generation of tracers or novel tracers that have been tested. In fact, promising results have been reported about Tau PET imaging, including evidence that Tau tracers’ uptake is more closely related to neurodegeneration diagnostic biomarkers than to the presence of amyloid [67]. In addition, Tau PET biomarkers may work best before NFTs are prevalent, supporting the early identification of tauopathies [68,69]. Concerning genetic aspects related to neurodegeneration and dementia, post-mortem evaluation reported a strong correlation between Tau aggregates and Tau PET findings in MAPT mutation carriers ([^18^F]AV-1451 PET study) [70]. Similarly, a case-control study described how Tau PET shows a higher magnitude of binding in MAPT mutation carriers who harbor mutations that are more likely to produce an AD-like Tau pathology, supporting the notion that this Tau PET ligand has specific binding properties for an AD-like Tau pathology [71]. Moreover, the presence of APOE4 seems to affect the Tau PET findings by promoting a more medial temporal lobe-predominant pattern of Tau pathology [72].

The complexity of Tau deposits, including their heterogeneous histopathology, composition and conformations, explains the discussion about the binding spectrum/sites of Tau PET tracers and the elaboration of novel PET tracers [73]. In recent years, several PET tracers targeting abnormal conformations of the Tau protein have been developed, which have allowed researchers to visualize Tau aggregates in vivo [74]. Several first-generation Tau PET tracers for imaging Tau deposits, such as the [^11^C]PBB3, [^18^F]Flortaucipir (also known as T807 or [^18^F]-AV1451) and [^18^F]THK arylquinoline series were evaluated in humans and have been developed to assess the neurofibrillary pathology in vivo. In addition, several research institutes and pharmaceutical companies have been working on developing second-generation Tau PET tracers with a higher binding affinity and selectivity [75] to overcome the limitations of the first-generation Tau PET tracers. So far, PET imaging with first-generation Tau tracers has been carried out in primary tauopathies. [^18^F]AV1451, a tracer of the [^18^F]THK family, and [^11^C]PBB3 have, in the CBS/PSP spectrum, shown the regional pattern of Tau pathology expected in these diseases with relatively good discrimination from healthy volunteers [67].

### 3.1. First-Generation Tracers

Several Tau PET tracers have been tested in humans. However, most of these radiotracers encountered some major limitations and have not been fully validated yet. Tauvid ([^18^F]-Flortaucipir) was approved by the U.S. Food and Drug Administration (FDA) in 2020 for PET imaging of adult patients with cognitive impairments undergoing evaluation for Alzheimer′s disease (AD) based on Tau pathology [76].

#### 3.1.1. [^18^F]FDDNP (C_18_H_16_FN_3_)

A naphthylethylidene derivative, [^18^F]FDDNP, was the first PET tracer to visualize the Alzheimer’s disease pathology in living humans [77]. FDDNP is not an exclusive Tau marker. In fact, it binds to both neurofibrillary tangles and amyloid plaques in the brains [75]. The FDDNP retention has been described in several types of diseases including prion disease, frontotemporal dementia, Down’s syndrome, progressive supranuclear palsy and also in American football players with suspected chronic traumatic encephalopathy (CTE) [74,78,79,80]. However, there is some conflict between PET findings and in vitro binding data [74]. 

#### 3.1.2. [^11^C]PBB3 (C_17_H_15_N_3_OS)

A pyridinyl-butadienyl-benzothiazole derivative, [^11^C]PBB3, has been developed as a unique chemotype-class Tau PET radiotracer [74], and was reported to detect a broad range of Tau deposits. [^11^C]PBB3 imaging clearly differentiates AD from healthy controls [81] and it is also a useful tool in non-AD dementia, such as CBD [81,82]. [^11^C]PBB3, on the other hand, is light sensitive and its radioactive metabolite can enter the brain, which complicates the quantification of the Tau pathology [83]. In addition, [^11^C]PBB3 imaging correlates with long-term neuropsychiatric outcomes in patients with traumatic brain injury, with promising results both in distinguishing patients with traumatic encephalopathy syndrome from patients without traumatic encephalopathy syndrome and in determining the severity of the disease [84]. Radioligand binding to brain homogenates revealed multiple binding components and indicated distinct selectivity of PBB3 compared to AV-1451 for diverse Tau fibril strains. This highlights the more robust ability of PBB3 to capture wide-ranging Tau pathologies [85].

#### 3.1.3. [^18^F]T808 (C_17_H_19_FN_4_)

A benzo[4,5]imidazo[1,2-a]pyrimidine derivative, [^18^F]T808, has been reported as a selective Tau tracer [73]. The target of [^18^F]T808 (similarly to [^18^F]T807) is aggregates of hyperphosphorylated Tau (PHF). Although [^18^F]T808 showed favorable kinetic properties, this agent encountered some limitations due to the high bone uptake, which revealed the problem of defluorination [76,86].

#### 3.1.4. THK Arylquinoline Series

[^18^F]-THK523-(C_17_H_15_FN_2_O).

[^18^F]-THK5351-(C_18_H_18_FN_3_O_2_).

Novel quinoline derivatives were initially identified as candidate Tau PET tracers by the screening of over 2000 small molecules [87], with the initial selection of the [^18^F]THK523 agent, which failed to visualize the Tau deposits clearly in patients with AD [74]. In addition, quinolone-based tracers such as [^18^F]THK523 showed high nonspecific binding in the white matter [75]. Compound optimization of arylquinoline derivatives resulted in the development of a further three [^18^F] labeled radiotracers: [^18^F]THK5105, [^18^F]THK5117 and [^18^F]THK5351. These compounds possess higher binding affinities for Tau aggregates in AD brains than [^18^F]THK523, and have preferable pharmacokinetics without defluorination in vivo [88]. However, all these tracers also showed substantial binding in areas not primarily related to the accumulation of the Tau pathology in AD (e.g., the basal ganglia) [89]. For tracers of the THK arylquinoline aeries, the signal in the basal ganglia has been preliminarily attributed to binding to monoamine oxidase B (MAO-B) [90,91].

#### 3.1.5. [^18^F]Flortaucipir (Tauvid) (C_16_H_10_FN_3_)

Tauvid is an [^18^F] labeled benzimidazole pyridine derivative, selected and developed by screening a varied chemical class of compounds using isolated PHF-Tau from postmortem AD brain tissues and intact human brain tissue sections [92]. Tauvid is also known as [^18^F]Flortaucipir, [^18^F]AV-1451 and [^18^F]T807 [76].

A multicenter study compared the brain distribution and retention of [^18^F]Flortaucipir to florbetapir PET, also considering the amyloid status, type of diagnosis, age and cognitive function, and supported the hypothesis that the Aβ and Tau pathology may start independently [33]. If confirmed, this hypothesis may represent a crucial aspect of the AD neuro-physiopathology that may influence future research in the AD and dementia field, with a potential impact on future perspectives concerning novel therapeutic approaches and the personalized management of the patient. In addition, the hypothesis of an independent start of the Aβ and Tau pathology will corroborate the importance of developing an accurate tool for the in vivo detection of tauopathy, which may provide complementary information with respect to amyloid pathology in vivo assessment.

Furthermore, they corroborate the potential association between cortical Tau, cognitive impairment and neuronal dysfunction. In addition, the localization and magnitude of cortical atrophy in an AD post-mortem case-series was also associated closely with [^18^F]Flortaucipir findings [93].

A further paper supported the accuracy and reliability of [^18^F]Flortaucipir for PET scan interpretation [94], and in addition, [^18^F] Flortaucipir PET imaging could predict the rate of brain atrophy as determined by structural MRI [95]. However, some limitations of [^18^F] Flortaucipir concern progressive supranuclear palsy (PSP). Even if Tauvid imaging is useful when comparing PSP and PD patients [96], there is a substantial overlap of signals in multiple brain regions when comparing PSP patients with AD patients and healthy subjects [97].

Similar limitations have been described in patients with CTE, Down’s syndrome and dementia with Lewy bodies [67,76,98,99].

When compared to the other first-generation Tau tracers, Tauvid (or [^18^F]Flortaucipir) overcame some limitations, and therefore, the agent received approval from the FDA for clinical usage. However, Tauvid PET imaging is still facing several challenges (particularly off-target binding), which may limit its application in clinical use. The clinical indication of Tauvid is for estimating the density and distribution of NFTs in the brains of adult patients with cognitive impairments who are being evaluated for AD by PET [76].

### 3.2. Tau PET Tracers’ Distribution Pattern 

#### 3.2.1. Distribution Pattern of [^18^F]Flortaucipir

Most of the studies published in the Tau PET tracers field have concerned [^18^F] Flortaucipir (FTP) PET findings and described distribution patterns that may support nuclear medicine physicians in the interpretation of PET data both in research and clinical fields. 

In either young or older cognitively normal volunteers, Jie et al. described little focal cortical retention of [^18^F]Flortaucipir [76]. In addition, elderly cognitively normal volunteers showed frequent retention in the mesial temporal lobes, and some also in the brainstem or striatum [100]. The increased tracer retention in regions of the medial temporal lobe in elderly subjects predicted a worse episodic memory performance [101,102].

A recent paper focused on the Tau tracer distribution in the healthy elderlyand observed that Tau accumulation occurred in anatomically specific patterns, in contrast to the more diffuse nature of Aβ deposition (evaluated by using PiB PET). Schöll et al. demonstrated the ability to segregate participants based on [^18^F]Flortaucipir accumulation in specific Braak stages and described differences in the patterns associated with age and Aβ [101]. 

A further paper employed an unsupervised data-driven method to identify spatial patterns of Tau-PET tracer distribution and compared these patterns to previously published “pathology-driven” ROIs. By using this data-driven approach, images were entered into a robust voxelwise stable clustering algorithm, which resulted in five clusters, suggesting that [^18^F]Flortaucipir PET data naturally cluster into spatial patterns that are biologically meaningful and that may improve and support the clinical management of the AD patient [102]. A further paper evaluated the performance of several methods to obtain parametric images of [^18^F]Flortaucipir [103]. Golla et al. concluded that RPM (receptor parametric mapping) and SA (spectral analysis) provided parametric images comparable to the non-linear regression estimates. However, individual SUVratio values were biased compared with the distribution volume ratio. The bias in the SUVratio for a specific scan interval was not constant but appeared to depend on the underlying Tau load. It was relatively constant in healthy controls (∼10%) for different scan intervals, whereas in the case of AD, not only was a higher bias observed but also greater variability. In addition, the impact of regional blood flow changes on the SUV ratio or PET indices, for several reasons, needs further investigation. Therefore, further studies in larger samples are needed in order to clarify these aspects because quantification is essential in clinical practice, especially in longitudinal studies and in treatment evaluation. The SUVratio, meanwhile, due to the reported high variability, may provide erroneous results. In addition, kinetic modeling of [^18^F]Flortaucipir has been described in healthy and AD subgroups, with no univocal results. However, this interesting paper, aimed at evaluating the potential role of parameters derived from kinetic analysis with the SUV ratio, did not take into account other crucial factors, such as the SUV ratio reproducibility at different time intervals or its sensitivity to blood flow (especially considering regional blood flow alterations due to multiple causes, or the potential impact of changes in permeability or blood-brain barriers alterations on the SUV ratio and PET parameters). Further papers in larger samples are needed to clarify the potential role of the kinetic approach in the Tau PET field [104].

Concerning the cortical regions affected in the AD spectrum, in subjects with mild cognitive impairment and AD, retention seems to spread from the mesial temporal lobes to isocortical areas, consistent with the Braak staging of neurofibrillary Tau pathology [76,100]. In addition, in MCI/AD patients, [^18^F]Flortaucipir binding in entorhinal, limbic and neocortical regions is associated with cortical atrophy. When quantifying [^18^F]Flortaucipir uptake across brain lobes, both local and distant associations with grey matter atrophy have been observed. Particularly, in a previous paper, the association between [^18^F]Flortaucipir findings and atrophy was described in ROI Braak III–IV and Braak V–VI, but not in ROI Braak I–II [105]. 

A further paper confirmed that Tau tracer signal patterns in the cognitively unimpaired correspond to the early Braak stage but also suggest tangle involvement in extra-medial temporal and extra-temporal regions that are considered more advanced in the Braak scheme, even when amyloid negative [106]. Distinct patterns of neurofibrillary tangle deposition in early-onset Alzheimer′s disease dementia versus late-onset Alzheimer′s disease dementia provide evidence of variability in regional tangle deposition patterns. Lowe et al. reported that different disease phenotypes have different patterns of tauopathy, suggesting the possibility of widespread development of an early tangle pathology rather than a pattern defined exclusively by adjacent, region-to-region spread.

In a recent paper, Sonni et al. developed a visual interpretation method for [^18^F]Flortaucipir Tau-PET and tested it on 274 individuals (including controls, MCI, AD, non-AD) [107]. A global visual score was described (0 = no binding; 1 = mild binding; 2 = intense binding) in seven cortical regions. Moreover, Sonni et al. elaborated four [^18^F]Flortaucipir distribution patterns, constituting a promising approach to Tau measurement in clinical practice.

— Pattern I (negative scan): absence of [^18^F]Flortaucipir signal in any brain area;
— Pattern II (mild temporal binding only): mild to moderate increase of [^18^F]Flortaucipir signal, limited to the medial temporal cortex and fusiform gyrus, probably consistent with early Braak stage Tau pathology in older individuals with or without cognitive decline [11,27,30];
— Pattern III (AD-like binding): [^18^F]Flortaucipir distribution not localized only in the medial temporal/fusiform area (extension to lateral temporal, parietal or frontal cortices) consistent with the neuropathological distribution of Tau in advanced Braak stage [11,27,30];
— Pattern IV (non-AD-like): atypical [^18^F]FTP signal distribution, not following the expected Braak distribution of Tau tangles (for example, predominant white matter or subcortical binding consistent with non-AD syndromes) [107].

This approach seems promising in the clinical management of the patient with dementia and may be useful, especially in the differential diagnosis. However, this visual analysis also has a potential role in providing a novel insight into the preclinical tauopathy assessment and early diagnosis. As an emerging tool, further papers are needed in order to demonstrate the accuracy of [^18^F]FTP imaging in tauopathy assessment, and subsequently, in dementia management. It will be interesting to evaluate the potential predictive role of [^18^F]FTP imaging throughout the AD continuum and in other neurodegenerative diseases, with a special focus on MCI conversion and early detection of preclinical conditions.

An interesting paper described regional patterns of Tau tracer uptake on PET imaging in atypical AD variants: posterior cortical atrophy (PCA) and logopenic progressive aphasia (lvPPA) [108]. Both syndromes showed diffuse Tau tracer uptake throughout all cortical regions, although PCA showed greater uptake in occipital regions compared to lvPPA. The Tau pattern reported in lvPPA showed greater uptake in left frontal and temporal regions compared to PCA and predominant left-asymmetric Tau deposition. The PCA Tau tracer pattern was mainly bilateral. Tetzloff et al. also described a regional Tau tracer uptake correlation with age (younger subjects showed greater Tau tracer uptake bilaterally in the frontal and parietal lobes than older subjects) and with clinical findings. 

A further paper demonstrated that [^18^F]Flortaucipir Tau imaging—contrary to amyloid-beta imaging—shows a strong regional association with clinical and anatomical heterogeneity in AD [109]. In fact, Ossenkoppele et al. reported different Tau PET tracer patterns associated with clinical presentation:

— Patients with an amnestic-predominant presentation (*n* = 5) showed the highest [^18^F]Flortaucipir retention in medial temporal and lateral temporoparietal areas;
— Patients with lvPPA (*n* = 5) demonstrated asymmetric left- greater than right-hemisphere [^18^F]Flortaucipir uptake in three of five patients;
— In most AD patients who underwent all three PET exams, there was a strong negative association between [^18^F]Flortaucipir and [^18^F]FDG uptake and less pronounced positive associations between [^11^C]PiB and [^18^F]FDG and [^18^F]Flortaucipir and [^11^C]PiB;
— In all patients, younger age was associated with greater [^18^F]Flortaucipir uptake in wide regions of the neocortex, while older age was associated with increased [^18^F]Flortaucipir in the medial temporal lobe.

Non-amnestic (or atypical) presentations of neurodegenerative dementias are generally underrecognized and underdiagnosed. According to the abovementioned findings, [^18^F]FTP imaging can support the differential diagnosis of neurodegenerative causes of dementia. In addition, [^18^F]FTP uptake seems consistent with the expected distribution of neurological changes in atypical presentations of AD: the shreds of evidence collected by using [^18^F]FDG imaging demonstrate different hypometabolic patterns according to the clinical heterogeneity of AD presentation, with an asymmetric left predominant pattern in lvPPA and a posterior hypometabolic pattern in PCA [66]. Future papers may provide interesting correlations between tauopathy, atrophy, hypometabolism pattern and clinical presentation in the dementia field. If confirmed by multicenter trials in larger samples, the assessment of tauopathy using Tau PET imaging may improve the patient management and our knowledge concerning the neurological alterations beyond the disease, including a view of the timeline of neurological changes in atypical presentations of AD.

However, according to the recent network degeneration hypothesis, the stereotypical anatomical propagation of the Tau pathology is thought to be indicative of misfolded Tau proteins, also possibly spreading along functional cortical networks. This hypothesis is still a matter of debate, but a recent Tau PET study elaborated a methodology aimed to assessing if Tau pathology accumulation should occur in functionally connected brain regions [110]. Whitwell et al. evaluated if the Tau-dependent seed-based networks corresponded with known functional resting-state networks and examined the relevance considering Braak staging. The authors identified 10 independently coherent Tau pathology networks, with the majority showing symmetrical bi-hemispheric expansion and corresponding to highly functionally connected brain areas (such as the precuneus and cingulate cortex). A set of independently coherent Tau pathology networks were reported in AD patients, associated with the severity of disease, and consistent with functional networks previously reported to be impaired in the AD neuropathology.

In a further previous paper focused on tauopathy distribution in cognition-relevant networks in AD, the authors quantified the spatial correspondence of the regional distribution pattern of PET-evidenced Tau pathology in AD with functional brain networks (by using large-scale resting state functional magnetic resonance imaging data) in two independent AD and prodromal AD published datasets. In the BioFINDER dataset (https://biofinder.se/, accessed on 24 August 2021), the typical AD Tau tracer distribution pattern (involved predominantly inferior, medial and lateral temporal cortical areas, as well as the precuneus/posterior cingulate and lateral parts of the parietal and occipital cortex) overlapped primarily with the dorsal attention, and to some extent with higher visual, limbic and parts of the default-mode network. In the ADNI sample (more prodromal group), the overlapping was less pronounced but showed a highly similar distribution pattern, suggesting an earlier stage of the previously described pattern. In conclusion, the study indicates that the regional deposition of Tau aggregates in AD predominantly affects higher-order cognitive over primary sensory-motor networks but does not appear to be specific to the default mode or related limbic networks [111].

Furthermore, similarly to THK arylquinoline series, concerns have been raised about substantial off-target binding, in particular to the enzyme monoamine oxidase B (MAO-B), which may limit routine clinical applications [67,112]. The exact contribution of MAO-B binding to the total off-target signal, and the brain areas that are particularly vulnerable to this off-target signal, remain to be determined for the available Tau tracers. 

Therefore, the development of Tau PET imaging and the improving properties of novel tracers may provide novel insight into the network degeneration hypothesis, by reporting possible correlations between Tau accumulation and neurodegeneration signs along with functional cortical networks. This may also provide an overview of the neurodegenerative steps of the neurological changes in AD, both in a regional environment and in a network environment. Due to the crucial role of Tau accumulation in the AD pathology, the in vivo assessment of tauopathy may provide novel information regarding the network degeneration hypothesis and/or further hypotheses in the dementia field.

A further paper reported the assessment of PSP changes by using FTP PET imaging. The FTP uptake was elevated in PSP versus controls and PD patients in a pattern consistent with the expected distribution of the Tau pathology. The biodistribution pattern described in clinical PSP included bilaterally elevated FTP uptake in the globus pallidus, putamen, subthalamic nucleus, midbrain and dentate nucleus relative to controls and PD patients. In addition, globus pallidus binding best distinguished PSP patients from controls and PD (area under the curve (AUC)  =  0.872 vs. controls, AUC  =  0.893 vs. PD) [96]. Further studies are needed in order to assess the role and the specific indications of Tau imaging in primary tauopathies and further neurodegenerative causes of dementia. However, this initial description of expected tracer distribution patterns in each disease may support the clinical management of the patient and the research field in dementia. In addition, Tau PET imaging may provide novel biomarkers and parameters in differential diagnosis in the dementia field.

#### 3.2.2. Distribution Pattern of Other Tau Tracers

[^11^C]PBB3 clearly differentiated AD brains from healthy control brains, and tracer retention in the hippocampus confirmed the binding affinity to NFTs. In AD patients, specific [^11^C]PBB3 binding was observed in the CA1 and subiculum regions in the hippocampus, where a high density of fibrillar Tau aggregates exists [79]. High binding of [^11^C]PBB3 was also detected in the medial temporal lobes, precuneus and frontal cortex in AD. In addition, a case with corticobasal syndrome (CBS) showed elevated [^11^C]PBB3 retention in the basal ganglia, and the spatial pattern of PBB3 retention was consistent with that of brain atrophy [81].

Recent longitudinal studies of [^18^F]THK5117 demonstrated that annual changes in tracer binding were significantly increased in the temporal cortex of patients with AD, which was closely associated with the annual changes in cognitive decline [113]. Recent [^18^F]THK5351 PET studies demonstrated greater specific signals in the frequent sites of Tau deposition and lower non-specific binding in the white matter than [^18^F]THK5117 [114]. In a comparative study between [^18^F]THK5117 (Tau) and [^11^C]PiB (Aβ) PET, Li et al. observed that on average Tau tracer positive-voxels were closer to the white matter than the Aβ tracer positive voxels in all AD subjects and for all regions, both before and after regionally adjusting for the non-specific white-matter binding of both tracers. The differential laminar pattern was validated post-mortem [115]. However, previous research suggested different molecular targets for these tracers. According to a previous paper, while [^11^C]PBB3 appeared to preferentially bind to Tau deposits with a close spatial relationship to amyloid-beta, the [^11^C]THK5351 pattern was more consistent with the expected distribution of the Tau pathology and to clinical findings of AD [116].

A further paper compared PET scans with [^18^F]THK5317 and [^18^F]FDG at the baseline and follow-up, neuropsychological assessment at the baseline and follow-up and a scan with [^11^C]PIB (amyloid-β deposition) at the baseline only in AD and CBD patients. The pattern of changes in [^18^F]THK5317 retention was heterogeneous across all patients, with qualitative differences both between the two AD groups (prodromal and dementia) and among individual patients, highlighting that heterogeneous Tau tracer uptake among patients with symptomatic AD, in contrast to the homogeneous changes seen in glucose metabolism, better tracked clinical progression [117].

A further biodistribution pattern has been described in PSP with [^11^C]PBB3 PET imaging, supporting the role of Tau tracers in the differential diagnosis and in the management of different neurodegenerative causes of dementia. PSP patients exhibited greater radioligand retention than healthy control subjects in multiple brain regions, including the frontoparietal white matter, parietal gray matter, globus pallidus, STN, red nucleus and cerebellar dentate nucleus. In addition, [^11^C]PBB3 deposition in the frontoparietal white matter was correlated with the general severity of Parkinsonian and PSP symptoms, whereas both gray matter and white matter tracer accumulations in the frontoparietal cortices were associated with nonverbal cognitive impairments [118], supporting the promising role of Tau PET imaging in tauopathy assessment.

### 3.3. Second-Generation Tracers and Future Perspective 

Studies regarding first-generation tracers led to the approval of Tauvid and the first generation of Tau PET tracers are now used in clinical research; however, concerns have been raised about off-target binding and low sensitivity. A lack of correlation between pathological Tau load and tracer binding has been reported, in addition to a limited sensitivity to Tau in early disease, and high variability in tracer binding between and within cases [119]. A new generation of Tau PET tracers has recently been developed with the additional aim of overcoming some of the limitations associated with the MAO-B affinity and improving the pharmacokinetic profile. These new radiotracers have been optimized to have a high affinity for Tau neurofibrillary tangles, which are composed of paired helical filaments (PHFs) and are characteristic of Alzheimer’s disease [112]. In fact, the dynamic range that may differentiate the second-generation tracers from the first-generation tracers is related to several pharmacological property factors, including pharmacokinetics, the ability to enter the brain structures and the chemical and metabolic stability.

Therefore, the scientific community has been working on developing second-generation Tau PET tracers that should overcome some limitations of the first-generation tracers [75,76], particularly the “off-target” binding that interferes with the quantification of [^18^F]Flortaucipir in several brain regions. A previous paper compared [^18^F]Flortaucipir with the novel Tau tracer [^18^F]RO948 head-to-head in vivo. [^18^F]RO948 and [^18^F]Flortaucipir bound comparably in neocortical regions, but [^18^F]RO948 showed higher retention in the medial temporal lobe. [^18^F]RO948 also presented lower intracerebral “off-target” binding [120].

A novel, promising isoquinoline derivative with high affinity and selectivity for Tau aggregates is JNJ-64326067, which could be useful for the detection and quantitation of Tau aggregates [121]. 

Further multicenter trials are needed in order to define the properties of novel compounds. In particular, further papers may provide useful data concerning the biochemical properties of Tau tracers in AD and other tauopathies, along with defining the differences and characteristics of each tracer. However, one of the most promising second-generation tracers, already tested in clinical scenarios, is [^18^F]MK-6240. Considering the Braak-stage classification, [^18^F]MK-6240 imaging provides an index of early and late Tau accumulation as well as the disease stage in preclinical and symptomatic individuals. These characteristics support the potential role of this second-generation tracer in the management of living patients with Alzheimer′s disease in the near future [122,123], showing favorable kinetics as well [124]. A further quantitative study paper described results from serial arterial blood samples in order to obtain metabolite-corrected input functions. Following intravenous administration of [^18^F]MK-6240, the tracer rapidly partitioned into the brain and its heterogeneous distribution pattern was consistent with the known NFT pathology in AD, suggesting that [^18^F]MK-6240 has adequate SUVR T-RT characteristics and supporting the use of this outcome in future studies [125].

Beyond the crucial potential role in AD, few data have been produced concerning Tau imaging with second-generation tracers in further neurodegenerative diseases. Yet, an interesting case-report on the distribution pattern of [^18^F]MK-6240 in CTE [126] suggested a potential role of Tau imaging in detecting and distinguishing this neurodegenerative disorder from further diseases in the dementia field.

A further promising second-generation tracer is [^18^F]PM-PBB3, which has been tested both in AD and in primary neuropathies with promising results [67], including FTD [127]. In fact, tauopathies are characterized by the deposition of Tau fibrils composed of conformers specific to each illness, and therefore, different tracers may reflect the heterogeneity of Tau deposition. As reported in a previous study, distinct selectivity of different tracers for diverse Tau fibril strains has been described [85]. In FTD patients, [^18^F]PM-PBB3 in the left frontal lobe overlapped with the hypometabolic region detected by FDG PET. However, a comparison of FTP and [^18^F]PM-PBB3 in FTD patients with MAPT mutations described different findings based on the Tau conformers (3R/4R and 4R), reporting promising results concerning the in vivo detection of both 3R/4R and 4R Tau domains with [^18^F]PM-PBB3 [127]. In fact, if a panel of radiochemicals has enabled in vivo assessment of tauopathy in AD, a similar result has not been achieved in further tauopathies. A recent study aimed to capture different Tau deposits, in order to focus on further tauopathies, especially FTD [128], also demonstrating the binding of [^18^F]PM-PBB3 to Tau deposits in FTD in murine models. Then, a clinical study has been performed describing increased binding of [^18^F]PM-PBB3 in dementia patients, reflecting cortical-dominant AD and subcortical-dominant progressive supranuclear palsy (PSP) Tau topologies. In addition, the in vivo reactivity of [^18^F]PM-PBB3 with FTLD Tau inclusion was strongly supported by neuropathological examinations of brains derived from Pick′s disease, PSP and corticobasal-degeneration patients who underwent Tau PET imaging [128].

Promising results have been reported with the usage of [^18^F]PI-2620 PET. Initial clinical data obtained in AD and HC subjects demonstrated a high image quality and excellent signal-to-noise ratio in AD subjects [129]. Furthermore, a multicenter evaluation based on post-mortem autoradiography indicates a value of [^18^F]PI-2620 to differentiate suspected patients with PSP, potentially facilitating a more reliable diagnosis of PSP [130]. For [^18^F]PI-2620, it has been also shown that early-phase images can be used as a surrogate of neuronal injury in tauopathies or overlapping Parkinsonian syndromes (Alzheimer′s disease, progressive supranuclear palsy, corticobasal syndrome, multi-system atrophy, Parkinson′s disease, frontotemporal dementia). Therefore, dynamic imaging or a dual-time-point protocol for Tau-PET imaging could supersede additional [^18^F]FDG-PET imaging by indexing both the distribution of Tau and the extent of neuronal injury. However, as also described for other Tau tracers, the potential impact of changes of blood flow in quantitative parameters is still a matter of debate. Therefore, the data obtained from the early or dynamic acquisition should be interpreted with caution as they may reflect information about the cerebral blood flow and metabolism instead of the Tau pathology [131]. 

Several trials are ongoing and novel Tau PET tracers are under testing, as listed in Table 1.

## 4. Conclusions

The advance of in vivo Tau imaging has provided promising results in the dementia field, especially in AD. In vivo Tau imaging could support the management of dementia patients and could serve as a biomarker both in clinical practice and in the research field [67]. In addition, Tau PET imaging may support differential diagnosis in the dementia field, which represents a challenge [3,132]. Tau tracers have been tested in clinical settings in several neurodegenerative diseases, including classic AD and atypical AD presentations, PSP, FTD, CTE, etc. However, the studies regarding Tau PET tracers in primary tauopathies and dementia are still ongoing. In the future, further information can be provided by clinical and preclinical studies. In fact, a limitation of the present study is the lack of a comprehensive report about preclinical studies, especially for second-generation tracers. A future goal can be the evaluation of preclinical data on novel tracers, with a special focus on binding properties that may differ in different kinds of Tau deposits, with a potential crucial impact on differential diagnosis in the dementia field and tauopathies. 

As previously described, Tauvid received approval from the FDA for clinical usage, but this agent is still facing several challenges, in particular off-target binding, which may limit its application in clinical use [76]. In particular, the off-target binding in the first-generation tracers (both THK family and Tauvid), especially in the basal ganglia, has been attributed to the binding to monoamine MAO-B [90,91,112]. Due to the great interest demonstrated by the scientific community, the second-generation tracers should overcome these limitations and should allow for a better in-vivo assessment of Tau deposition [75,76]. Beyond their accuracy, the second-generation Tau tracers have been produced to present more favorable pharmacokinetic properties and a more favorable dynamic range, including a higher affinity to neurofibrillary tangles, which should improve tauopathy assessment. Several clinical trials are ongoing but the actual knowledge about second-generation tracers is promising. The possible applications of Tau PET imaging range from diagnosis to disease monitoring, considering the closer correlation with neuronal dysfunction as compared with amyloid imaging [67]. Description of distribution patterns of Tau tracers, especially regarding the approved Tauvid, may support nuclear medicine physicians in the interpretation of Tau PET data in clinical practice, especially considering the off-target binding in the white matter. In particular, the recent approach proposed by Sonni et al. provides a visual interpretation method for Tau-PET. The description of four distribution patterns—Pattern I (negative scan), Pattern II (mild temporal binding only), Pattern III (AD-like binding), Pattern IV (non-AD-like) [106]—seems promising in describing the heterogeneity of tauopathy distribution (and subsequently Tau tracers’ uptake) in the dementia field. In fact, Tau PET imaging may support the management of dementia patients in several neurodegenerative causes of dementia, including in the case of atypical presentation of AD. This method has been applied to Tauvid analysis, and a similar visual interpretation method can be extended to the novel tracers after further papers are published. 

A weakness of this study is the relative lack of available data about distribution patterns of second-generation tracers since several studies are still ongoing. Therefore, a future goal may be the integration of data about the biodistribution of second-generation tracers, in order to provide a comprehensive review that may support nuclear medicine physicians in the visual interpretation of PET data. A novel insight may also be provided by the evaluation of differences in the biodistribution of PET tracers in different neurodegenerative causes of dementia. In addition, further papers may provide novel information about the acquisition methodology, including eventual information about dynamic acquisitions or comparison of early and late acquisitions. 

However, besides clinical support with the management of the patient, a possible role of Tau PET imaging in the dementia field, especially in AD, is the improvement of knowledge concerning the neuro-physiopathology, which may provide novel insight, especially in the development of future therapies. For this purpose, the integration of preclinical data, especially concerning novel tracers, may provide further useful information. Tauopathy assessment may support the personalized clinical management of the patient, providing further data that may influence the management and the therapy of the patient. In addition, future novel treatments based on the inhibition of Tau aggregation may require in vivo tauopathy assessment (both in the testing and monitoring phases). Indeed, Tau PET imaging could support the discovery of and serve as a therapy monitoring tool in novel treatments linked to NFL metabolism (ClinicalTrials.gov Identifier: NCT02791191), and eventually, Tau deposition.

In this review, we described the Tau deposition assessment by using Tau PET imaging, and especially, the distribution patterns. However, an interesting aspect that can be explored by using Tau PET tracers is the network degeneration hypothesis [110]. This is still a matter of debate but the quantification of connectivity alterations and the consequent derivation of individual metrics are matters of great importance in the field of neurodegenerative disease [133]. Therefore, the functional evaluation of associative brain areas, also with [^18^F]FDG imaging, in patients affected by Alzheimer′s disease is still a matter of debate [134]. In this context, as demonstrated in a study by Yang et al., the association of Tau PET imaging and structural connectivity graphs may be useful to predict the Tau spread in brain areas at a certain time-point [135]. The promising role of this association has been demonstrated in other important studies by Franzmeier et al. [136,137]. These aspects should be explored in further papers in larger samples, but a deeper evaluation of network degeneration represents a future goal of Tau PET imaging.

Tau PET imaging is a promising tool, both in research and clinical practice, in the dementia field.

## Figures and Tables

**Table 1:** An overview of Tau tracers from the study in clinical trials, as registered on the ClinicalTrials.gov platform (https://clinicaltrials.gov/; last accessed on 24 August 2021).

Tau Tracer	Chemical Formulas	ClinicalTrials.gov Identifier	Title	Condition or Disease
*[^18^F]RO6958948*		*NCT03174938*	*The Swedish BioFINDER 2 Study*	*Dementia, AD, PD, Lewy body disease, etc.*
		*NCT02792179*	*Evaluation of [^18^F] RO6958948 as Tracer for Positron Emission Tomography (PET) Imaging of Tau Burden in Alzheimer′s Disease Participants*	*AD*
		*NCT02187627*	*Evaluation of [^11^C]RO6924963, [^11^C]RO6931643, and [^18^F] RO6958948 as Tracers for Tau Imaging with Positron Emission Tomography in Healthy Control Subjects and Subjects with Alzheimer′s Disease*	*Healthy controls, AD*
*^11^C-RO6924963;* *^11^C-RO6931643*		*NCT02187627*	*Evaluation of [^11^C]RO6924963, [^11^C]RO6931643, and [^18^F] RO6958948 as Tracers for Tau Imaging with Positron Emission Tomography in Healthy Control Subjects and Subjects with Alzheimer′s Disease*	*Healthy controls, AD*
*[^18^F]PM-PBB3*	*C_20_H_20_FN_3_O_2_S*	*NCT04248270*	*A Novel Tau Tracer in Young Onset Dementia*	*AD, Vascular dementia, dementia*
		*NCT03625128*	*[^18^F]PM-PBB3 PET Study in Tauopathy Including Alzheimer′s Disease, Other Dementias and Normal Controls*	*AD, CBS,* *Frontotemporal Dementia, PSP, etc.*
		*NCT04169126*	*Topography Staging and Dual Phase Image Quantification of Tau PET in Cognitive Impairment Subjects*	*AD*
*[^18^F MK-6240*	*C_16_H_11_FN_4_*	*NCT04104659*	*Study of Tau Imaging with the Use of [^18^F]MK-6240 Tracer*	*AD*
		*NCT03860857*	*MRI and PET Biomarkers for Cognitive Decline in Older Adults*	*AD, Cognitive* *impairment and decline*
		*NCT03706261*	*Alzheimer′s PET Imaging in Racially/Ethnically Diverse Adults*	*AD*
		*NCT04784416*	*Transcranial Photobiomodulation for* *Alzheimer′s Disease (TRAP-AD)*	*MCI, AD*
		*NCT03071224*	*Phase 1 Evaluation of [^18^F]MK-6240 PET as an Imaging Marker for Tau Protein*	*AD, Healthy*
		*NCT04437290*	*University of Washington Alzheimer′s Disease Research Center (UW ADRC) Imaging & Biomarker Core*	*AD*
*[^18^F]PI2620*	*C_15_H_9_FN_4_*	*NCT04715750*	*Evaluation of Imaging Characteristics of [^18^F]PI-2620 PET in AD and PSP Patients Using High and Low Specific Activity*	*AD, PSP*
		*NCT04566003*	*Evaluation Comparing Two Tau PET Radiotracers, [^18^F]PI-2620 and [^18^F]GTP1, in Subjects With Normal Condition or Prodromal to Moderate Alzheimer′s Disease*	*AD*
*[^18^F]GTP1*		*NCT04566003*	*Evaluation Comparing Two Tau PET Radiotracers, [^18^F]PI-2620 and [^18^F]GTP1, in Subjects With Normal Condition or Prodromal to Moderate Alzheimer′s Disease*	*AD*
		*NCT02640092*	*Longitudinal Evaluation of [^18^F]GTP1 as a PET Radioligand for Imaging Tau in the Brain of Participants With Alzheimer′s Disease Compared to Healthy Participants*	*AD*
*[^18^F]MNI-958*	*C_20_H_20_FN_3_O_2_S*	*NCT03545789*	*Phase 1 Test-retest Evaluation of [^18^F]MNI-958 PET*	*Healthy, AD, PSP*
		*NCT03058965*	*Phase 0 Evaluation of [^18^F]MNI-958 as a Potential PET Radioligand for Imaging Tau Protein in the Brain*	*Healthy, AD, PSP*
*[^18^F]MNI-952*		*NCT03080051*	*Evaluation of [^18^F]MNI-952 as a Potential PET Radioligand for Imaging Tau Protein in the Brain*	*Healthy, AD, PSP*
*[^18^F]MNI-1020*		*NCT03239561*	*Evaluation of Tau Protein in the Brain of Participants with Alzheimer′s Disease Compared to Healthy Participants*	*AD*
